# A reproducible, high throughput method for fabricating fibrin gels

**DOI:** 10.1186/1756-0500-5-423

**Published:** 2012-08-08

**Authors:** Kaitlin C Murphy, J Kent Leach

**Affiliations:** 1 Department of Biomedical Engineering, University of California, Davis, Davis, CA, 95616, USA; 2 Department of Orthopaedic Surgery, School of Medicine, University of California, Davis, Sacramento, CA, 95817, USA; 3 Department of Biomedical Engineering, University of California, Davis, 451 Health Sciences Drive, Davis, CA, 95616, USA

**Keywords:** Fibrin, Hydrogel, Polydimethylsiloxane, Mesenchymal stem cells, Bone

## Abstract

**Background:**

Fibrin gels are a promising biomaterial for tissue engineering. However, current fabrication methods are time intensive with inherent variation. There is a pressing need to develop new and consistent approaches for producing fibrin-based hydrogels for examination.

**Findings:**

We developed a high throughput method for creating fibrin gels using molds fabricated from polydimethylsiloxane (PDMS). Fibrin gels were produced by adding solutions of fibrinogen and thrombin to cylindrical defects in a PDMS sheet. Undisturbed gels were collected by removing the sheet, and fibrin gels were characterized. The characteristics of resulting gels were compared to published data by measuring compressive stiffness and osteogenic response of entrapped human mesenchymal stem cells (MSCs). Gels exhibited compressive moduli nearly identical to our previously reported fabrication method. Trends in alkaline phosphatase activity, an early marker of osteogenic differentiation in MSCs, were also consistent with previous data.

**Conclusions:**

These findings demonstrate a streamlined approach to fibrin gel production that drastically reduces the time required to make fibrin gels, while also reducing variability between gel batches. This fabrication technique provides a valuable tool for generating large numbers of gels in a cost-effective manner.

## Findings

### Background

Fibrin gels are a promising biomaterial, as they can be delivered in a minimally invasive manner or formed into an implant and tuned to possess desired characteristics (*e.g.*, gelation rate, compressive stiffness, degradation rate, *etc.*) [1-3]. These biodegradable hydrogels are produced through the combination of proteins found in the body, fibrinogen and thrombin, which generates a provisional matrix that allows cells to encounter an environment that closely mimics the ECM of a damaged tissue [4,5]. A wide variety of methods are available to manipulate the physical properties of these gels, including increasing the protein concentrations [6], cross linking *via* enzymes or UV radiation [7], and adjusting the Ca^2+^ concentration [8-10]. Recently, we showed that supplementing the pre-gel solution with various concentrations of sodium chloride (NaCl) alters gel stiffness [11]. This approach addresses many shortcomings of other fibrin gel fabrication techniques; namely, that one can manipulate gel stiffness using biomimetic concentrations of fibrinogen and thrombin while maintaining a slow gelation time, thus limiting stress on entrapped cells [12].

Previous work in our laboratory fabricated fibrin gels by combining solutions of fibrinogen and thrombin in parafilm-covered, cylindrical nylon washers [11]. Once gelation was complete, the gels were carefully removed with a metal spatula and placed in tissue culture plates. This method was taxing to reproduce, required significant preparation, and was time intensive, requiring more than 12 hours, on average, to produce up to 30 gels. Poly-dimethylsiloxane (PDMS) is a chemically inert, hydrophobic, silicone-based organic polymer that can be produced with a wide range of viscosities. High viscosity PDMS is often used in microfluidic devices, and while pliable, it will retain its form indefinitely. These properties make PDMS an ideal material for creating a non-fouling, biologically inert substrate for use in fabricating fibrin gels [13,14].

This study demonstrates a novel method for producing fibrin gels using a PDMS mold. Compared to previous methods, these gels can be fabricated in a high throughput and cost-effective manner, thus providing an improved technique for generating reproducible substrates for use in studies of materials science, cellular biology, and tissue development.

### Methods

#### Fabrication of PDMS mold

Cylindrical molds were formed using a 6 mm diameter biopsy punch (Accuderm Inc., Ft. Lauderdale, FL, USA) pressed into a 2 mm thick sheet of PDMS (Dow Corning, Midland, MI, USA). The PDMS mold can be used for up to six months. To initiate gel fabrication, the PDMS mold was rinsed with distilled water, sterilized by immersion in 70% ethanol for up to 2 min, and allowed to dry in an aseptic environment for 5 min. When dry, the PDMS sheet was placed in a non-treated sterile polystyrene dish, creating a seal.

#### Fibrin gel preparation

**Figure 1:**
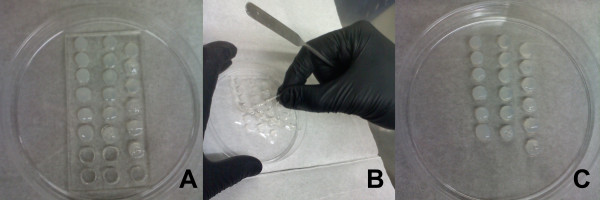
Gel solution was added to each cylindrical mold, the contents were allowed to gel for 1 hr (1A), then the PDMS sheet was carefully removed (1B), leaving behind undisturbed fibrin gels (1C).

Fibrin gels were prepared by combining 20 mg mL^-1^ fibrinogen (Calbiochem, Gibbstown, NJ, USA), 1.16-3.85% (w/v) NaCl (Sigma Aldrich, St. Louis, MO, USA), 2.5 U mL^-1^ thrombin (Calbiochem), 20 mM CaCl_2_ (Sigma Aldrich), and 250 KIU mL^-1^ aprotinin (Santa Cruz Biotechnology, Inc., Santa Cruz, CA, USA), all in PBS. A total volume of 80 μL was added to each cylindrical mold, and the contents were allowed to gel for 1 hr in standard culture conditions (Figure 1A). The PDMS sheet was then carefully lifted from the culture dish (Figure 1B), leaving behind the undisturbed fibrin gels (Figure 1C), and the gels were transferred to 12-well tissue culture plates. This fabrication method requires 4 hours to produce up to 100 gels.

#### Compressive testing of fibrin gels

We measured the compressive moduli of gels made with varying salt concentrations to verify that fibrin gels fabricated in this manner possessed comparable mechanical properties to those prepared using previously published methods. Acellular gels containing increasing NaCl content were allowed to gel for 1 hr, transferred to PBS and allowed to swell for 1 hr, and then any excess fluid was blotted off before analysis. Gels were then loaded between two flat platens and compressed at 1 mm/min (Instron 3345 Compressive Testing System, Norwood, MA, USA). The compressive moduli were measured from the 0-5% linear regions of the stress–strain graphs [4].

#### Cell culture and osteogenic response

We analyzed the osteogenic response of human mesenchymal stem cells (MSCs) entrapped within fibrin gels as a measure of comparison with previously reported fabrication methods. Human MSCs (Lonza, Walkersville, MD, USA) were expanded in αMEM (Invitrogen, Carlsbad, CA, USA) supplemented with 10% fetal bovine serum (JR Scientific, Woodland, CA, USA) and 1% penicillin/ streptomycin (Mediatech, Manassas, VA, USA) until use at passage 6. MSCs were added to the pre-gel solution at 1.5 × 10 [6] cells mL^-1^ of gel solution and cultured for various time intervals in the αMEM media described above.

At days 3, 5, 7, and 10, the gels were collected and sonicated in 400 μL passive lysis buffer (Promega, Madison, WI). Samples were centrifuged at 5000 rpm for 10 min to pellet the cell debris, and the supernatant was collected. The supernatant was analyzed for intracellular alkaline phosphatase (ALP) activity using a *p*-nitrophenyl phosphate (PNPP) colorometric assay as previously described [11]. DNA content, a measure of cell growth, was assayed from the supernatant using the Quant-iT PicoGreen dsDNA Assay Kit (Invitrogen).

#### Statistical analysis

Results are expressed as mean ± standard deviation (SD). Statistical analyses were performed by ANOVA followed by Student–Newman–Keuls *post-hoc* tests assessing significance to probability values (*p*) <0.05.

### Results and discussion

#### Material characterization

**Figure 2:**
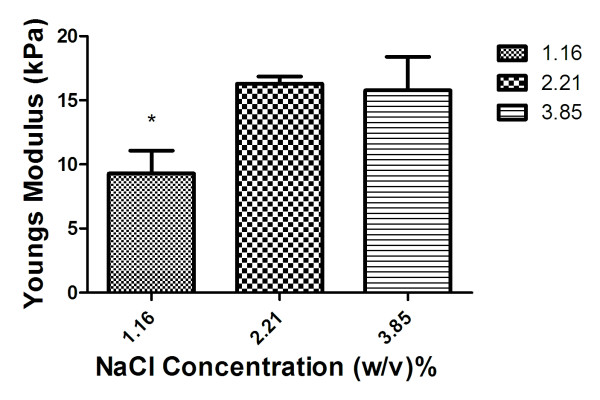
** Young’s modulus of acellular fibrin gels made in PDMS molds.** Data are mean ± SD (n = 9).

The compressive stiffness of fibrin gels with increasing NaCl content were determined and compared to previously reported values. Similar to gels formed in nylon washers, fibrin gels with increasing NaCl content exhibited increasing Young’s modulus up to 2.21% (w/v) NaCl, above which the modulus began to decrease, on average (Figure 2). Experimentally determined values of gel stiffness were similar to previously reported values of stiffness for gels produced in the nylon washers (Table 1). These data demonstrate that this high throughput method yields fibrin gels with similar mechanical properties to those produced with the nylon washer technique.

**Table 1:** Comparison of compressive stiffness between two fabrication methods

**Concentration (w/v)%**	**PDMS Mold**		**Nylon Washer**		
	**Mean (kPa)**	**St Dev (kPa)**	**Mean (kPa)**	**St Dev (kPa)**	**Percent Difference**
1.16%	9.29	1.78	12.56	1.53	26.01%
2.21%	16.29	0.57	16.64	1.89	2.10%
3.85%	15.78	2.61	17.24	2.85	8.49%

#### Osteogenic response of entrapped MSCs

**Figure 3:**
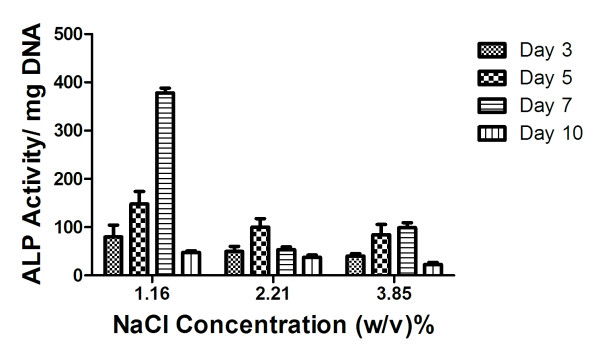
** Intracellular ALP activity of MSCs entrapped in fibrin gels.** Data are mean ± SD (n = 9). *p < 0.05 vs. other groups.

We measured ALP activity of MSCs entrapped in the fibrin gels formed with the addition of 1.16, 2.21, or 3.85% (w/v) NaCl. We observed the expected trend of temporal protein activity in each group, with ALP activity peaking on different days as a function of gel composition. ALP activity followed a similar trend to previously published values for MSCs suspended in fibrin gels with similar NaCl concentrations formed within nylon washers (Figure 3). These data provide another indication that cells behave similarly when entrapped in fibrin gels formed in either PDMS molds or nylon washers.

### Conclusions

We developed a reproducible, cost-effective, and quick method for fabricating fibrin gels using molds formed in PDMS. This new fabrication method generated fibrin gels with similar compressive moduli and the capacity to promote osteogenic differentiation of entrapped MSCs. Values from these two characterization assays were similar to our previous data, thus validating this new approach for the consistent and reproducible production of many hydrogels in a fraction of the time required for previous methods.

## Competing interests

The authors have no financial or non-financial competing interests related to this study.

## Authors’ contributions

KCM designed, carried out, and analyzed the study. JKL designed the study and edited the manuscript. Both authors read and approved the final manuscript.
